# Investigating the Risk Factors in Progression of HIV Disease Using an Illness-Death Multistate Model

**DOI:** 10.34172/jrhs.11310

**Published:** 2025-09-15

**Authors:** Roghayyeh Hassanzadeh, Hossein Mahjub, Mohammad Mirzaei, Fariba Keramat, Maryam Farhadian

**Affiliations:** ^1^Department of Biostatistics, School of Public Health, Hamadan University of Medical Sciences, Hamadan, Iran; ^2^Student Research Committee, Hamadan University of Medical Sciences, Hamadan, Iran; ^3^Research Center for Health Sciences, Institute of Health Sciences and Technologies, Avicenna Health Research Institute, Hamadan University of Medical Sciences, Hamadan, Iran; ^4^Department of Epidemiology, School of Public Health, Hamadan Health Center, Hamadan University of Medical Sciences, Hamadan, Iran; ^5^Department of Infectious Diseases, School of Medicine, Hamadan University of Medical Sciences, Hamadan, Iran; ^6^Brucellosis Research Center, Hamadan University of Medical Sciences, Hamadan, Iran

**Keywords:** HIV/AIDS, Survival analysis, Multistate model, Antiretroviral therapy, Tuberculosis

## Abstract

**Background:**

The trend of human immunodeficiency virus (HIV) disease progress is different for every patient. Some patients may experience events during the course of their disease that can affect disease progression and death. The main objective of the present study was to investigate the effect of risk factors in progression of HIV disease, taking into account intermediate events, using a multistate model.

**Study Design:**

A retrospective cohort study.

**Methods:**

The current study used information from 673 HIV-infected adult patients registered at the Hamadan Provincial Health Center in Iran, between 1997 and 2023. A multistate framework was described to investigate the progression of HIV disease over time. Three states (HIV-infected, acquired immunodeficiency syndrome [AIDS], and death) and three possible transitions (from HIV to AIDS, from HIV to death, and from AIDS to death) were considered in this framework. An illness-death multistate model was applied to determine the effect of risk factors on these transitions.

**Results:**

The results revealed that receiving antiretroviral therapy (ART) significantly decreased the hazard of transition from HIV to AIDS, whereas older age, tuberculosis (TB) co-infection, and treatment with the final guideline intensified the hazard of the mentioned transition. Low education, older age, and unprotected sexual transmission increased the risk of transition from HIV to death, while receiving ART and treatment with the final guideline decreased the risk of this transition. Receiving ART, being employed, having a history of prison, and being treated with the final guideline could decrease the hazard of transition from AIDS to death, whereas TB co-infection increased the hazard of this transition.

**Conclusion:**

Implementing strategies for early diagnosis, timely treatment, adherence to treatment, as well as screening and TB treatment, especially at younger ages, can be useful in reducing AIDS progression and mortality.

## Background

 Despite significant progress in prevention and treatment, the human immunodeficiency virus/acquired immunodeficiency syndrome (HIV/AIDS) is still a major global public health concern. To date, around 88.4 million people have been infected with HIV, and around 42.3 million have died worldwide as a result of HIV. In 2023 alone, nearly 1.3 million people were infected with HIV, and 630,000 people died as a result of HIV.^1^ Despite global efforts to end the AIDS epidemic by 2030, the current rate of reduction is insufficient.^2^ As in other parts of the world, there are also many cases of HIV in Iran. According to the latest data from 2023, 42,000 adults were infected with the virus, 2,500 new cases were reported, and 1,900 died of AIDS.^3^

 Although there is currently no definitive cure for HIV, prevention and control of the disease are possible with antiretroviral therapy (ART), a widely used medical treatment.^1^ Treatment with ART has been shown to significantly reduce viral replication while improving the performance of the immune system, thereby improving the quality of life and dramatically increasing life expectancy.^4-6^ It also plays a crucial role in HIV/AIDS prevention and can help slow down the progression of AIDS and reduce the risk of mortality.^6-11^ Without ART, most HIV-infected people develop AIDS within 5–10 years, and in some cases even faster, within only 3–5 years, and usually survive only 2–3 years after AIDS diagnosis.^12,13^

 However, the progression of HIV disease in HIV-infected patients can vary due to immunological, genetic, environmental, and virological factors.^14,15^ The treatment process can also be complicated by some factors, including chronic conditions associated with immunodeficiency, chronic viral and bacterial infections, and HIV drug resistance.^10,16-18^ In addition, co-infection with opportunistic infections, such as tuberculosis (TB) and hepatitis, as well as poor adherence to treatment and self-medication due to drug-drug interactions, can increase the risk of death and AIDS.^10,15,17,19-21^ In this way, the disease can progress to AIDS in some people but not in others. Patients who progress to the AIDS stage have a higher risk of death, and it is important to identify the prognostic factors that influence a patient’s progression to AIDS. Moreover, the development of AIDS can alter the progression of HIV disease, and the effect of prognostic factors can be altered by AIDS. As a result, the effects of the variables on time to AIDS onset and time to death with and without AIDS may be different. Therefore, special statistical methods are required to determine the prognostic factors that influence the survival of these patients.

 A HIV-positive patient may experience an intermediate event, such as progression to AIDS and then death, or may die without such progression.^10,11,22^ This is an important aspect that has often been neglected in many HIV/AIDS survival studies, and separate analyses have been conducted for each endpoint.^16,23-25^ In these analyses, the relationships between these events could not be taken into account. The neglect of intermediate states and their time of occurrence can also lead to biased or misleading results.^10,11^ A common approach to this problem is to use multistate models that model different types of events simultaneously. Multistate models describe the progression of the disease and the transitions between the different stages over time and provide a better understanding of the impact of prognostic factors on disease progression and outcomes.^26-28^ Therefore, this study aims to investigate the effect of risk factors on progression to AIDS and death, both with and without progression to AIDS, using an illness-death multistate model.

## Methods

###  Data description

 A retrospective cohort study was conducted using data from HIV-infected patients registered at the Hamadan Provincial Health Center in Iran between 1997 and 2023. The main dataset contained 730 patients who were at least 15 years old and had a confirmed diagnosis of HIV infection. In the present article, based on the multistate model, 57 patients who had AIDS at the time of study entry were excluded from the main dataset, and the analysis was performed on the remaining 673 patients.

 A person with HIV infection was considered HIV positive, regardless of clinical stage, if confirmed by laboratory criteria according to the definitions and requirements of each country.^30^ In Iran, a person is considered HIV-positive if, until a few years ago, they had two positive enzyme-linked immunosorbent assay (ELISA) tests and a positive Western blot, or, more recently, a rapid HIV test followed by two positive ELISA tests, one of which is a fourth-generation test.^31^ In addition, according to the World Health Organization, an HIV case was considered an AIDS case if diagnosed with a stage 4 condition, either presumptive or definitive, and/or if their CD4 count dropped below 200 per mm³ of blood.^30^

 Baseline demographic and clinical information was extracted from patients’ medical records using a predefined checklist, including gender (female/male), age ( ≤ 30, 31-40, 41–50, and > 50 years), marital status (married, single, and other), education level (high [high school to academic]/low [illiterate to middle school]), and occupational status (employed/unemployed). Other related data were the treatment guideline used (initial guideline, intermediate guideline, and final guideline), history of drug abuse (yes/no), history of imprisonment (yes/no), mode of HIV transmission (injecting drug user, unprotected sexual and unknown), ART status (yes/no), and TB infection (yes/no). Based on data registered by the Ministry of Health and Medical Education, we just specified the status of covariates at the time of HIV diagnosis.

 The treatment guideline was defined using the variables according to the treatment guidelines in force at the time of HIV diagnosis: the initial guideline (before 2006), the intermediate guideline (2006–2011), and the final guideline (after 2011). The main differences between these guidelines concern the timing of ART initiation: the initial and intermediate guidelines recommended ART initiation based on specific CD4 cell counts, whereas the final guideline recommends ART initiation immediately after HIV diagnosis, regardless of CD4 count.

**Figure 1:**
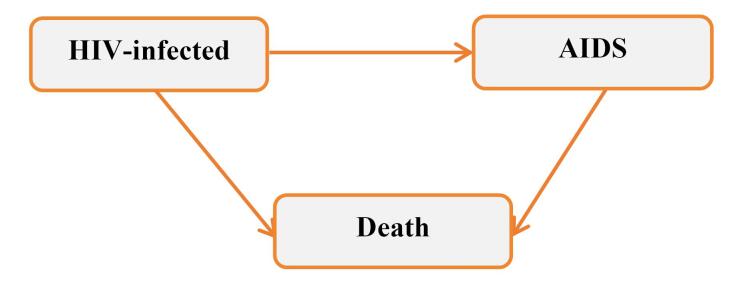
The Multi-State Structure of HIV Patients. *Note*. HIV: Human immunodeficiency virus; AIDS: Acquired immunodeficiency syndrome

 The study focused on two main endpoints: AIDS and death. The outcomes of interest were the times of entry into each state (in months), including time to AIDS progression, time to death (without AIDS progression), and time to death after AIDS progression. All patients who were alive at the end of the study were censored for the death endpoint, and those who did not progress to AIDS were also censored for the AIDS event. Additionally, patients who were lost to follow up or withdrew from the study were considered censored. The multistate structure of the data is illustrated in Figure 1.

###  Multistate model

 Multistate models are used to describe the progression of disease over time using a set of states and transitions between them. In these models, different types of events (including final and intermediate events) are modeled simultaneously. Each model is defined by states and transitions. States represent the status of a subject at a given time, while transitions refer to the movement from one state to another. There are three different types of states (initial, transient, and absorbing). An initial state is the point at which a subject enters the process, and a transient state is a state that a subject can enter and exit at any time. An absorbing or final state is one that the subject cannot leave once it is entered. Unlike absorbing and initial states, transient states can be revisited multiple times during the course of the study. In these models, both transient and absorbing states can be considered outcomes, and each possible transition between states is treated as an event. Each possible transition is associated with a transition hazard, representing the instantaneous risk of moving from one state to another at a specific time. In multistate models, transition hazards, called transition intensities, are modeled, and the effects of covariates on them are assessed.^26-28^

 An HIV-infected individual may experience death either after progression to AIDS or without developing AIDS. For this reason, in this study, the progression of HIV was described by incorporating an intermediate state (AIDS), and an illness-death multistate was considered with three states: HIV infection (the initial state), AIDS, and death (the absorbing state), and three transitions: from HIV infection to AIDS, from AIDS to death, and from HIV infection to death (Figure 1). A transition-specific Cox model was fitted to investigate the effects of covariates on these transition hazards. The Markov property is assumed, implying that the transition hazard at any given time depends solely on the individual’s current state rather than on their history. The mathematical definition of the model is as follows:


(1) $$\lambda_{ij}(t\left|Z\right.)=\lambda_{ij,0}(t)\;\exp\left\{\beta_{ij}^{T}Z_{ij}\right\}$$

where *λ*_ij,0_ (t) is the baseline intensity function for the transition from state *i* to state *j* at time t. ***β***_ij_ represents the vector of regression coefficients, and ***Z***_ij_ denotes the vector of covariates specific to the transition from state *i* to state *j*.

 Maximum likelihood estimation was used to estimate the regression coefficients of the model. Then, the effect of a covariate, such as ***Z***_ij,p_ on the transition intensity of a specific transition i→j was measured using the hazard ratio, which is defined as exp (*β*_ij,p_).

 It should be noted that the Markov assumption was checked and confirmed. Further, to select the variables in the final model, a univariate analysis was applied, and a multistate model was fitted with a single variable. Next, the significance of each variable in all transitions was examined, and variables with a significance level less than 0.15 in at least one transition were included in the final multiple multistate models.

 All analyses were performed using “*Survival*”, “*mstate*”, and “*markovMSM*” packages of R software (version 4.2.2), with a significant level of 0.05.

## Results

 Of the 673 HIV-positive patients, 536 (79.6%) were male and 137 (20.4%) were female, with an age range of 18–85 years. The mean age ( ± standard deviation) of participants at the time of diagnosis was 35.80 ( ± 9.48) years, and 405 (60.1%) of them were treated according to initial and intermediate guidelines. Among patients, 41.3% and 53.3% were married and unemployed, respectively. Further, 42.6% were in the 31–40 age group, and 66.7% were infected with HIV through injection drug use. Furthermore, 82.8% had a low level of education. Nearly half of the patients (n = 347, 51.6%) received no ART, and 73.8% of them died. A small number of patients (n = 42, 6.2%) had co-TB. In addition, most patients had a history of imprisonment (n = 427, 63.4%) and a history of drug abuse (n = 514, 76.4%). Further details of participant characteristics are provided in Table 1.

**Table 1:** Demographic and clinical characteristics of HIV-infected patients in the total sample and event groups (AIDS and death) in Hamadan province

**Variables**	**Total, n=673**	**AIDS, n=275**	**Death, n=329**	**Median survival time (month)**	***P*** ** value**
Gender					0.001
Female	137	65	26	-	
Male	536	210	303	90	
Age (year)					0.005
≤ 30	210	85	118	105	
31-40	287	125	127	151	
41-50	124	44	65	73	
> 50	52	21	19	136	
Marital status					0.004
Married	287	101	112	172	
Single	239	101	146	80	
Other	156	73	71	152	
Education					0.056
High (high school to academic)	116	42	44	142	
Low (illiterate to middle school)	557	233	285	105	
Occupational status					0.227
Employed	314	115	142	119	
Unemployed	359	160	187	110	
Drug abuse					0.001
No	159	70	23	-	
Yes	514	205	306	87	
Imprisonment					0.001
No	246	105	79	183	
Yes	427	170	250	91	
Transmission way					0.001
Injecting drug user	449	186	281	86	
Unprotected sexual	184	73	40	-	
Unknown	40	16	8	-	
Antiretroviral therapy					0.001
No	347	74	256	52	
Yes	326	201	73	-	
Tuberculosis infection					0.034
No	631	235	300	118	
Yes	42	40	29	79	
Treatment guideline used					0.001
Initial guideline	143	48	112	66	
Intermediate guideline	262	123	159	99	
Final guideline	268	104	58	-	

*Note*. HIV: Human immunodeficiency virus; AIDS: Acquired immunodeficiency syndrome.

 The median follow-up time of the study was 69 months (interquartile range: 26–131.5). The mean and median survival times were 151.84 months and 112 months, respectively. Significant differences in survival were observed across the subgroups of all variables, except for occupational status and education (Log-rank test, Table 1).

 Based on the multistate structure (Figure 1), the number of direct transitions observed between the defined states during the study period is presented in Table 2. Among the 673 HIV-positive patients entered in the initial state, 275 (40.9%) progressed to AIDS, 220 (32.7%) died without progressing to AIDS, and 178 (26.4%) were censored for both progression to AIDS and death. Of the 275 patients who developed AIDS, 109 (39.6%) died, and 166 (60.4%) were censored for death following progression to AIDS. In total, 329 (48.9%) patients died, and 344 (51.1%) were censored during the follow-up period.

**Table 2:** Numbers of direct transitions between states during the study period in HIV-infected patients

**From**	**To**	**(1) HIV-infected**	**(2) AIDS**	**(3) Death**	**Total entering**
(1) HIV-infected		178	275	220	673
(2) AIDS		0	166	109	275
(3) Death		0	0	329	329

*Note*. HIV: Human immunodeficiency virus; AIDS: Acquired immunodeficiency syndrome.

**Figure 2:**
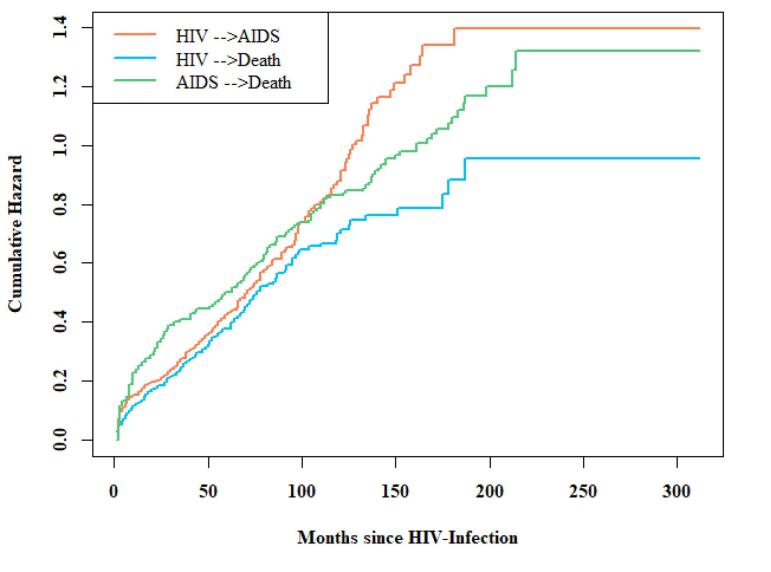
Estimated cumulative hazards of transition from HIV to AIDS, from HIV to death, and from AIDS to death in the multistate model. *Note*. HIV: Human immunodeficiency virus; AIDS: Acquired immunodeficiency syndrome

**Figure 3:**
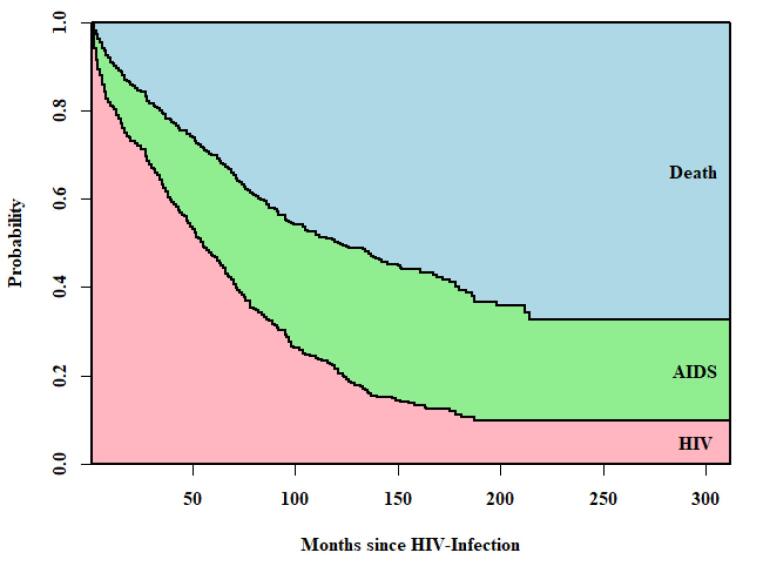
Stacked transition probabilities for HIV-infected patients. *Note*. HIV: Human immunodeficiency virus

 The cumulative hazard for each transition over time is depicted in Figure 2. The hazard of transition from HIV to death was consistently lower than that of HIV to AIDS throughout the study period. The hazard of transition from AIDS to death was the highest within the first 10 years, after which the hazard of transition from HIV to AIDS was high until the end of the study. In addition, the stacked transition probability plot is illustratedin Figure 3. In this plot, the vertical distance between two adjacent curves represents the probability of patients being in a specific state at a given time point. For example, 150 months after HIV-infection, the estimated probabilities of being in the HIV, AIDS, and death states were 0.15, 0.30, and 0.55, respectively.

 The results of the illness-death multistate model are reported in Table 3, demonstrating the effects of different covariates on transition hazards. According to the results, both ART and the year of HIV diagnosis were significantly associated with the intensities of all three transitions. Specifically, HIV patients who received ART had a significantly lower risk of both progression to AIDS (hazard ratio [HR] = 0.43, *P* < 0.001) and direct transition to death (HR = 0.05, *P* < 0.001) than those who did not receive ART. Additionally, among patients who progressed to AIDS, those who received ART had a lower risk of death (HR = 0.38, *P* < 0.001). Moreover, patients treated under the final guideline had a significantly higher risk of progression to AIDS (HR = 1.95, *P* < 0.001) compared to those treated under the initial guideline, but a lower risk of direct transition to death (HR = 0.55, *P* = 0.041). In patients who progressed to AIDS, patients treated with the final guideline also had a significantly lower risk of death than those treated with the initial guideline (HR = 0.51, *P* = 0.046).

**Table 3:** The effects of risk factors on transition risks in the illness-death multistate model: Progression from HIV-infected to AIDS and death (with and without progression to AIDS)

**Variables**	**HIV-Infection → AIDS**		**HIV-Infection → Death**		**AIDS → Death**	
	**HR (95% CI)**	***P *** **value**	**HR (95% CI)**	***P *** **value**	**HR (95% CI)**	***P *** **value**
Gender						
Female	1.00		1.00		1.00	
Male	1.02 (0.60, 1.74)	0.941	1.13 (0.52, 2.45)	0.759	1.63 (0.59, 4.52)	0.413
Age (year)						
≤ 30	1.00		1.00		1.00	
31-40	1.12 (0.84, 1.50)	0.446	1.17 (0.85, 1.61)	0.331	0.83 (0.52, 1.35)	0.457
41-50	1.59 (1.06, 2.38)	0.025	2.31 (1.55, 3.45)	0.001	1.29 (0.69, 2.43)	0.427
> 50	3.20 (1.87, 5.46)	0.001	4.23 (2.24, 7.99)	0.001	1.45 (0.45, 3.22)	0.712
Marital status						
Other	1.00		1.00		1.00	
Married	0.76 (0.56, 1.04)	0.090	0.88 (0.61, 1.28)	0.515	1.41 (0.77, 2.57)	0.267
Single	1.10 (0.78, 1.54)	0.600	0.93 (0.64, 1.34)	0.691	1.72 (0.98, 3.00)	0.059
Education						
High (high school to academic)	1.00		1.00		1.00	
Low (illiterate to middle school)	1.24 (0.89, 1.74)	0.212	1.98 (1.29, 3.06)	0.002	0.86 (0.50, 1.49)	0.589
Occupational status						
Unemployed	1.00		1.00		1.00	
Employed	0.84 (0.65, 1.10)	0.209	1.10 (0.83, 1.46)	0.503	0.52 (0.33, 0.82)	0.005
Drug abuse						
No	1.00		1.00		1.00	
Yes	1.00 (0.60, 1.67)	0.993	2.18 (0.97, 4.92)	0.060	1.84 (0.69, 4.98)	0.224
Imprisonment						
No	1.00		1.00		1.00	
Yes	0.91 (0.64, 1.30)	0.616	1.07 (0.74, 1.55)	0.704	0.57 (0.33, 0.97)	0.038
Transmission way						
Unknown	1.00		1.00		1.00	
Injecting drug user	1.29 (0.69, 2.40)	0.428	1.41 (0.50, 3.93)	0.514	1.70 (0.48, 6.04)	0.416
Unprotected sexual	0.84 (0.46, 1.55)	0.577	3.34 (1.10, 10.21)	0.034	1.33 (0.38, 4.62)	0.653
Antiretroviral therapy						
No	1.00		1.00		1.00	
Yes	0.43 (0.32, 0.58)	0.001	0.05 (0.03, 0.10)	0.001	0.38 (0.25, 0.58)	0.001
Tuberculosis infection						
No	1.00		1.00		1.00	
Yes	4.75 (3.32, 6.81)	0.001	0.19 (0.03, 1.37)	0.099	1.90 (1.14, 3.15)	0.013
Treatment guideline used						
Initial guideline	1.00		1.00		1.00	
Intermediate guideline	1.32 (0.93, 1.86)	0.116	1.15 (0.85, 1.56)	0.365	0.73 (0.44, 1.20)	0.216
Final guideline	1.95 (1.31, 2.89)	0.001	0.55 (0.34, 0.88)	0.014	0.51 (0.26, 0.99)	0.046

*Note*. HIV: Human immunodeficiency virus; AIDS: Acquired immunodeficiency syndrome; HR: Hazard ratio; CI: Confidence interval.

 Furthermore, older age at diagnosis significantly intensified the hazard of transition from HIV to AIDS and from HIV to death. The hazard of AIDS progression for HIV-positive individuals aged 41–50 and over 50 was 1.59 (*P* = 0.025) and 3.20 (*P* < 0.001) times that of those aged 30 years or less, respectively. Similarly, for patients in these age groups, the hazard of transition from HIV to death was 2.31 (*P* < 0.001) and 4.23 (*P* < 0.001) times that of those aged 30 years or less, respectively.

 HIV patients with TB were at a higher risk for transition from HIV to AIDS (HR = 4.75, *P* < 0.001) and from AIDS to death (HR = 1.90, *P* = 0.013) compared to those without TB infection. Likewise, the risk of direct transition to death was significantly higher among HIV patients with lower levels of education (HR = 1.98, *P* = 0.002) and those infected through unprotected sexual contact (HR = 3.34, *P* = 0.034).

 In addition, among AIDS patients, unemployed individuals had nearly twice the risk of death compared to employed ones (HR = 0.52, *P* = 0.005). Similarly, non-prisoners had a higher risk of death than prisoners in this group (HR = 0.57, *P* = 0.028).

## Discussion

 The present study described the progression of HIV disease using an illness-death model that encompasses the transitions from HIV to AIDS, from AIDS to death, and from HIV to death, and investigated the effect of several risk factors on the occurrence of these transitions. Of the risk factors examined in this study, ART and the applied treatment guideline significantly affected the hazard of all three transitions. Age at diagnosis and TB influenced the hazard of transition from HIV to AIDS. Education, mode of transmission, and age at diagnosis impacted the hazard of transition from HIV to death. Finally, occupational status and imprisonment, and TB affected the hazard of transition from AIDS to death.

 The findings of this study confirm that receiving ART had a key role in reducing the risk of HIV progression to AIDS, as well as the risk of death, both following AIDS and without its development. There were 57%, 95%, and 62% reductions in progression to AIDS, death without AIDS, and death after AIDS, respectively, compared to those who did not receive treatment. These findings highlight the strong protective effect of ART, consistent with the results of previous studies.^10,19,22,23,32,33^ Hence, early diagnosis and timely treatment should be prioritized to improve patient outcome.

 It was also found that patients treated with the final guideline had twice the risk of progressing to AIDS compared to those treated with the initial guideline, while their risk of death with or without AIDS was reduced by half. The higher AIDS progression for patients may be due to better screening and detection of HIV, even in late stages, along with delayed diagnosis, treatment initiation, or poor adherence to therapy when this treatment guideline was applied. However, improved care and easier access helped reduce the risk of death.^5,34-36^

 Moreover, the results indicated that patients co-infected with TB had a substantially higher risk of developing AIDS and dying from it, which is consistent with results of other studies.^10,11,16,23,25^ On the other hand, TB co-infection did not significantly affect the hazard of transition from HIV to death, which aligns with the findings of some studies while contradicting those of others.^9,10,13,19,32^

 Our findings revealed that patients with older age at the time of diagnosis had a higher risk for both HIV-to-AIDS and HIV-to-death transitions, which corroborates the results of many studies.^10,12,22,25,37-39^ They may be attributed to the association of older age with factors such as lower CD4 counts, more advanced disease stages, and delayed diagnosis.^5,40,41^

 In the present study, HIV patients with a lower level of education were more likely to experience direct death, which conforms to the findings of other studies.^13,42^ In addition, individuals infected through sexual transmission were at a higher risk of death without developing AIDS compared to those with unknown transmission. This difference may be due to individuals infected sexually being more likely to acquire co-infections, such as human papillomavirus, syphilis, candidiasis, and herpes, which weaken the immune system and increase the risk of death.^12,43^ However, this finding should be interpreted with caution, as individuals may have been exposed to multiple routes of transmission, and it is often impossible to accurately determine the primary route of transmission from the limited data recorded.

 In contrast to Cook et al,^44^ who reported no significant effect, the results of this study are consistent with those of Gheibi et al, demonstrating that employment was significantly associated with a reduced hazard of AIDS-related mortality.^38^ The findings also revealed a significant protective association between the risk of death and imprisonment among patients with AIDS, contrasting with the findings of Amiri et al^45^ Similar to previous studies, the reduced risk of death among prisoners in this study may be due to access to free and supervised ART, improved adherence, and reduced exposure to high-risk behaviors.^13,46^

 In this study, the cumulative risk of transition from AIDS to death was higher than that of transition from HIV to death, indicating that AIDS-related mortality occurred with greater intensity over time. However, our results also confirmed that deaths were more frequent overall in patients without AIDS progression. Before the implementation of the final guideline, mortality in this group was more than twice as high as in patients with AIDS; thereafter, the numbers were almost the same. This not only highlights the importance of timely ART initiation in HIV-positive patients without progression to AIDS but also emphasizes the need to focus more on non-AIDS-related causes of death in this population to effectively reduce overall mortality. Although we did not have data on the specific causes of death, the occurrence of mortality among patients without AIDS progression emphasizes the need for further investigation into non-AIDS-related factors.

 It should be noted that Hamidi et al applied a multistate model to analyze the HIV data from Hamadan. Their study was limited to data up to 2011 and included individuals of all age groups.^10^ In contrast, our study focused on adult patients and considered a longer follow-up period up to 2023, which allowed for an assessment of treatment guidelines and their impact on disease progression and mortality.

 This study had two limitations, as it used retrospective data collected in a health center. First, two important prognostic markers, baseline CD4 cell count and HIV viral load, were largely unavailable for patients diagnosed before 2011, because viral load was not measured at baseline according to the national guidelines at that time, and baseline CD4 count was not routinely assessed for most individuals referred during the early years of the outbreak. Second, some important factors, including late diagnosis, body mass index, treatment adherence, co-infection with hepatitis B or C, underlying diseases, and other opportunistic infections, were either not recorded or were incompletely documented, which could have affected HIV disease progression.

 It is worth noting that variables for the final multistate model were selected based on univariate multistate models. However, for future work, it is suggested to apply variable selection procedures for multistate data, such as the method proposed by Alafchi et al, to identify the most influential predictors.^47^

**Highlights**

- In this study, 329 patients died, of whom 109 (33%) died after progressing to acquired immunodeficiency syndrome (AIDS) and 220 (67%) died without AIDS progression. In other words, deaths without the progression of AIDS occurred more frequently.
- The cumulative hazard of transition from AIDS to death was higher than that from HIV to death.
- Receiving antiretroviral therapy (ART) significantly decreased the hazard of transition from HIV to AIDS, from human immunodeficiency virus (HIV) to death, and from AIDS to death.
- Patients aged 40 and over, with lower levels of education and unprotected sexual transmission, had a higher risk of transition from HIV to death.

## Conclusion

 In this study, the effect of prognostic factors on progression from HIV to AIDS and death, with or without developing AIDS, was assessed using a multistate model. The findings revealed that patients who received ART and were treated with the final guideline had a reduced risk of death, either with or without AIDS progression. TB intensified the risk of both AIDS progression and AIDS-related death. In addition, the cumulative hazard of AIDS-related death increased markedly over time, and the number of deaths directly attributable to HIV was high. These findings demonstrated that strict implementation of the ‘treat all HIV-positive patients’ strategy could be useful in reducing progression to AIDS and mortality. Furthermore, they highlighted the importance of implementation strategies for early diagnosis, timely treatment, and adherence to treatment, as well as screening and TB treatment, especially at younger ages, for both health providers and policymakers.

## Acknowledgements

 This work was a part of a PhD thesis in biostatistics submitted by Roghayyeh Hassanzadeh to Hamadan University of Medical Sciences, Iran. We would like to thank the Vice-Chancellor for Research and Technology of Hamadan University of Medical Sciences, Iran.

## Competing Interests

 The authors declare that they have no competing and conflict of interests.

## Ethical Approval

 This study was approved by the Research Ethics Committee of Hamadan University of Medical Sciences (IR.UMSHA.REC.1400.910), and the study complied with the relevant guidelines and regulations. Written informed consent was obtained from all participants; in the case of illiterate participants, it was obtained from their legal representatives with confidentiality regarding the patients’ names and surnames.

## Funding

 The study was supported by the Vice-Chancellor for Research and Technology, Hamadan University of Medical Sciences (Grant No. 1400120310201).
